# Research Review: A guide to computing and implementing polygenic scores in developmental research

**DOI:** 10.1111/jcpp.13611

**Published:** 2022-03-30

**Authors:** Andrea G. Allegrini, Jessie R. Baldwin, Wikus Barkhuizen, Jean‐Baptiste Pingault

**Affiliations:** ^1^ 4919 Division of Psychology and Language Sciences Department of Clinical, Educational and Health Psychology University College London London UK; ^2^ 4616 Social, Genetic and Developmental Psychiatry Centre Institute of Psychiatry, Psychology and Neuroscience King's College London London UK

**Keywords:** Polygenic scores, developmental research, longitudinal models

## Abstract

The increasing availability of genotype data in longitudinal population‐ and family‐based samples provides opportunities for using polygenic scores (PGS) to study developmental questions in child and adolescent psychology and psychiatry. Here, we aim to provide a comprehensive overview of how PGS can be generated and implemented in developmental psycho(patho)logy, with a focus on longitudinal designs. As such, the paper is organized into three parts: First, we provide a formal definition of polygenic scores and related concepts, focusing on assumptions and limitations. Second, we give a general overview of the methods used to compute polygenic scores, ranging from the classic approach to more advanced methods. We include recommendations and reference resources available to researchers aiming to conduct PGS analyses. Finally, we focus on the practical applications of PGS in the analysis of longitudinal data. We describe how PGS have been used to research developmental outcomes, and how they can be applied to longitudinal data to address developmental questions.

## Definition and calculation of polygenic scores

Since their conception and first application in human studies (Janssens et al., 2006; Purcell et al., 2009; Wray, Goddard, & Visscher, 2007), the use of polygenic scores in developmental research has become widespread. Polygenic scores (PGS) have become a standard downstream analysis in genome‐wide association studies (GWAS), and are widely employed by researchers in the behavioural, social and life sciences to predict complex traits and to infer genetic overlap between them. Different terms are typically used for PGS, including genetic (risk) scores (GRS), genome‐wide polygenic scores (GPS), polygenic indexes (PGI), or polygenic risk scores (PRS). These, however, broadly refer to individual scores based on measured genetic data [usually single nucleotide polymorphisms (SNPs)] conceptualized as indexes of the genetic predisposition, or burden, that an individual carries for a particular trait, disease or condition.

**Figure 1:**
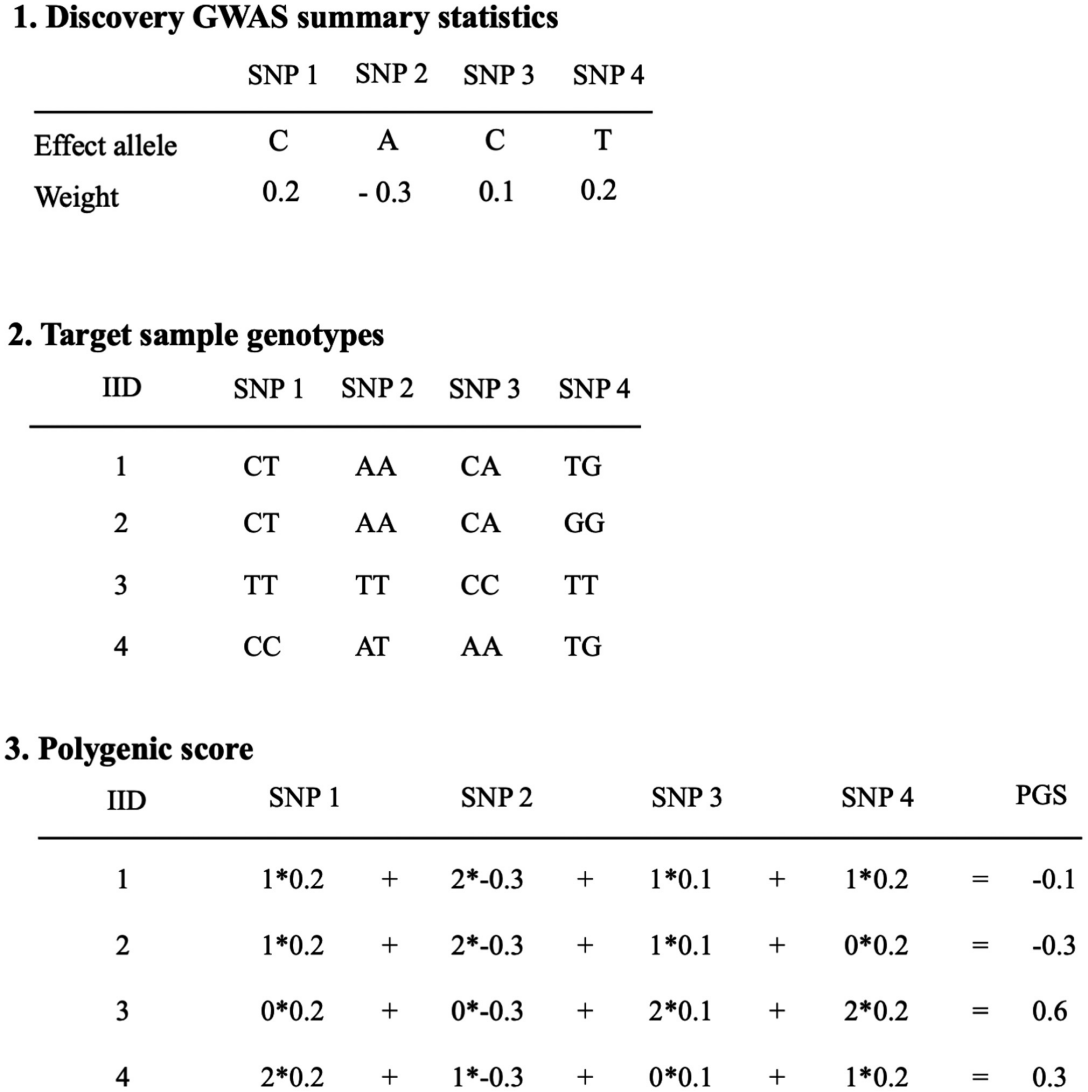
Basic stepwise process for calculating PGS. Effect sizes (weights) of single nucleotide polymorphisms (SNPs) are obtained from genome‐wide association studies (GWAS) in (large) discovery samples (Step 1). Based on the number of effect alleles carried by each genotyped individual in the target set (Step 2), a weighted sum is computed using the standardized estimate for each SNP in the discovery set multiplied by the number of effect alleles. A polygenic score can be computed based on just a few or on millions of SNPs, as in the case of genome‐wide polygenic scores. Computing a polygenic score results in a single value per individual and in a single variable per trait per sample

A basic stepwise process for calculating PGS is presented in Figure 1, key definitions are reported in Box 1. Formally PGS are defined as the linear combination $\hat{\text{PGS}}$ = **X** *b*, where **X** is an *n* × *p* matrix of *n* people by *p* SNPs, and *b* is a vertical vector of beta estimates for all SNPs, $b=(\beta_{1},\beta_{2},\ldots \beta_{p})$, obtained (typically) from external GWAS summary statistics.

$\hat{\text{PGS}}=\left[\begin{array}{ccc} {\text{snp}}_{11} & \cdots & {\text{snp}}_{1p}\\ \vdots & \vdots & \vdots \\ {\text{snp}}_{n1} & \cdots & {\text{snp}}_{\mathrm{np}} \end{array}\right]\left[\begin{array}{c} \beta_{1}\\ \vdots \\ \beta_{p} \end{array}\right]=\left[\begin{array}{ccc} {\text{snp}}_{11}*\;\beta_{1}+ & \cdots & +\;{\text{snp}}_{1p}\;*\;\beta_{p}\\ \vdots & \vdots & \vdots \\ {\text{snp}}_{n1}*\;\beta_{1}+ & \cdots & +\;{\text{snp}}_{\mathrm{np}}\;*\;\beta_{p} \end{array}\right]=\left[\begin{array}{c} {\text{PGS}}_{1}\\ \vdots \\ {\text{PGS}}_{n} \end{array}\right]$

That is, in their simplest form PGS are formulated as the weighted sum of trait‐associated alleles for a number of SNPs within an individual, ${\hat{\text{PGS}}}_{i}=\sum_{j=1}^{p}\left(x_{\mathrm{ij}}\beta_{j}\right)$, where $x_{\mathrm{ij}}\in \left\{0,1,2\right\}$ effect alleles for the *j*th SNP of the *i*th individual, that is, one row of the $\hat{\text{PGS}}$ matrix above (${\text{snp}}_{11}\;*\;\beta_{1}+\cdots +{\text{snp}}_{1p}\;*\;\beta_{p}$).

Box 1 **Definitions**

*Single nucleotide polymorphism (SNP)*: Common variation between individuals at a single position in the genetic code happening in at least 1% of the population.

*Cryptic relatedness*: Distant relationships between individuals that make people genetically similar, confounding associations in observational studies such as GWAS.

*Dominance*: Interaction between alleles within a genetic locus, that is, deviation from additivity within a locus.

*Epistasis*: Interaction of alleles across different genetic loci, that is, SNP–SNP interactions.

*Linkage disequilibrium*: the correlation between nearby variants on the same chromosome.

*Clumping*: Pruning of variants in linkage disequilibrium above a certain threshold (e.g. a correlation *r* > .1), prioritizing variants depending on a statistic of interest, typically *p*‐values in GWAS.

*Overfitting*: When a statistical model matches the data too closely modelling noise instead of the actual signal. This makes the model too optimistic and thus not generalizable well to independent data.

*Cross‐validation*: Resampling method used to evaluate a model within a unique dataset. The model parameters are learned in part of the dataset and performance is tested in a hold‐out set (e.g. with a 9 to 1 split for training and testing respectively).

*SNP‐heritability (SNPh^2^)*: Proportion of phenotypic variation explained jointly by all tagged SNPs.

By this definition of PGS, we assume that SNP effects act additively. This is a reasonable assumption, given that additive effects of common variants explain a substantial proportion of heritability in common complex traits (Yang et al., 2010). However, widespread epistatic effects (interactions between SNPs) are also likely to be at play (Huang & Mackay, 2016; Mackay & Moore, 2014). For disease risk models, we expect a nonlinear relationship between polygenic scores and the risk of disease, as the disease occurs only in the presence of a combined (high) load of risk variants at the individual level (Wray et al., 2021).

PGS are approximately normally distributed in the population with people varying on a continuum from low to high polygenic burden for a particular trait. The normal distribution of PGS is expected by the central limit theorem, as they reflect the summation of a large number of random variables. That is allele counts weighted by SNP effects for a particular trait.

### From GWAS to PGS

SNP weights used to derive PGS are typically obtained from external GWAS (discovery) samples. In GWAS, a phenotype vector *y* containing values for each *i*th individual in the sample is regressed on each measured *j* ^th^ SNP, additively coded for the number of minor alleles an individual carries (for example as 0 = CC, 1 = CT and 2 = TT, where T is the minor allele, that is, with the lowest frequency in the population), usually in the order of millions. For an *n* × *p* mean‐centered genotype matrix **X** containing genotype vectors of individual SNPs (*x*) for each person, the regression equation can be expressed as follows:

$$y=x_{j}\beta_{j}+\varepsilon$$

where $x_{j}$ is a vector of genotype values for individuals at the *j* ^th^ SNP, $\beta_{j}$ is the marginal effect for the *j* ^th^ SNP and ε the error term. This relationship is usually adjusted for demographic covariates such as age and sex, and a number of genetic principal components, to account for confounders such as population stratification and cryptic relatedness. In addition, depending on the cohort, technical confounders are accounted for, such as the version of genotyping chip used if different chip arrays were used to screen the cohort.

The marginal effects for the mean‐centered genotypes are given by:

$$\hat{\beta_{j}}=\frac{x_{j}^{t}y}{x_{j}^{t}x_{j}}=\frac{\text{cov}(x_{j},y)}{\text{var}(x_{j})}$$

where $x_{j}^{t}y/n=\text{cov}\left(x_{j},y\right)$ and $x_{j}^{t}x_{j}/n=\text{var}\left(x_{j}\right)$. Additive genetic variance at each locus is defined as $a^{2}=2pq\beta_{j}^{2}$, where 2*pq* is the variance of the genetic locus (the heterozygosity; with *p* and *q* the allele frequencies of a biallelic locus), and $\beta_{j}$ is the effect size obtained from the regression of the phenotype on the genotype as stated above. The proportion of variance explained in the phenotype can be calculated as $R^{2}=2pq\beta_{j}^{2}/y_{s^{2}}$, where $y_{s^{2}}$ is the variance of the phenotype ($R^{2}=2pq\beta^{2}$ for a standardized phenotype). Hence, the power to detect single SNP effects is a function of both the average genetic effect, and allele frequencies (Visscher & Goddard, 2019). Importantly, SNP‐trait associations are typically very small for common complex traits, with an inverse relationship between allele frequencies and SNP effect sizes (Park et al., 2011). Thus, the sample size of GWAS is of central importance for the discovery and estimation of SNP effects and, in turn, for the predictive power of PGS (Appendix S1) (Dudbridge, 2013).

**Figure 2:**
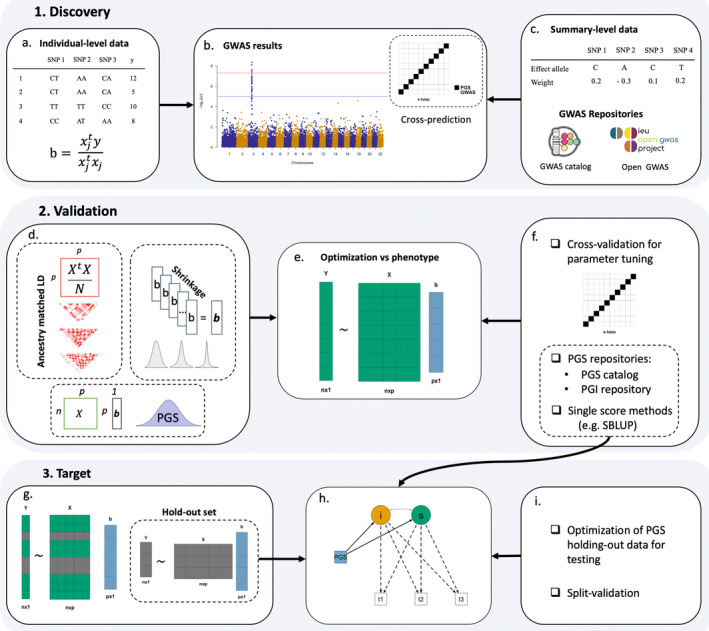
Figure PGS workflow. Marginal effects are obtained in the discovery set, either from individual‐level data (A) of large biobank‐scale studies, or from large GWAS metaanalyses (top panel). In a typical GWAS setting, weights are obtained by a series of regressions of a phenotype y on column vectors of a (in this case, mean‐centered) genotype matrix **X**, an *n* × *p* matrix of *n* observations by *p* SNPs (A). If sufficient sample size is available, a leave‐one‐out cross‐validation‐like approach (cross‐prediction) can be employed to obtain PGS in the discovery set by iteratively splitting the sample in k‐folds and leaving one part out for testing (B). Alternatively, researchers can leverage GWAS summary statistics from curated repositories, such as Open GWAS (C). Adjustment for ancestry matched linkage disequilibrium (LD; correlations between SNPs, X^t^X/N for a scaled genotype matrix, in Figure 2D) and optimization of tuning parameters (such as thresholding, or shrinkage parameters) versus a phenotype of interest (C and D) is then performed in the validation set. Here, (cross) validation is conducted to select the best combination of parameters to construct PGS (parameter tuning), before testing the performance of the optimal PGS in the target set. PGS are obtained by the linear combination of the (reweighted) vector of betas ***b*** with the genotype matrix **X** (D). Already calculated PGS from repositories (F), for example, LDpred‐based weights for a particular score, can be employed in this step instead of calculating the score from scratch (although validation will still need to be performed if multiple scores for a particular trait are available). Optimization can be performed in the target set directly via cross‐validation and holding‐out data for testing (G), or using split validation (I). Alternatively, pseudovalidation, single‐score methods (Table S1), or PGS weights from repositories, can be employed to directly obtain PGS in the target set where developmental models are fitted (H)

While we test for the effects of millions of variants on a phenotype, in practice, those tests are not independent due to linkage disequilibrium (LD). LD refers to the nonrandom association between nearby SNPs on a chromosome and is the reason for the typical association peaks in a ‘Manhattan plot’ (Figure 2B), which depicts association signals across the genome (i.e. the peak includes the causal SNP(s) and nearby SNPs in LD). Hence, the estimated GWAS marginal effects need to be adjusted for LD (depicted as a *p* × *p* correlation matrix in Figure 2D, more below).

We are thus conducting the equivalent of 1 million independent tests (Risch & Merikangas, 1996), leading to the typical genome‐wide significance threshold of 5 × 10^−8^ (i.e. 0.05/1 million; red threshold in Figure 2B). However, even if we impose such a stringent threshold to avoid false positives in a GWAS we do not need information about putative (causal) associations between SNPs and phenotypes to construct PGS. PGS including only GWAS significant variants are typically less powerful than PGS constructed using more lenient inclusion criteria. That is, PGS can handle false positives and up to a point there is a positive trade‐off, in terms of predictive power, between variants included in the score and noise added by the inclusion of false positives.

## Approaches to compute polygenic scores

### The traditional approach

Until recently, the standard way of constructing PGS was the clumping and thresholding (C + T) approach. It consists of performing an informed LD‐pruning (clumping) using *p*‐values from GWAS summary statistics to obtain a set of quasi‐independent SNPs prioritizing those most highly associated with the discovery trait. In a second step, SNPs above a particular GWAS *p*‐value threshold, for example, above the GWAS significant threshold of 5 × 10^−8^, are removed and a score is calculated with the remaining SNPs as described. The operation can be repeated with different thresholds generating different PGS including a different number of SNPs (e.g. using nominal significance, the blue line in Figure 2B).

The reason to perform clumping is that if SNPs in LD are included in the PGS without accounting for their correlation, the individual contribution of the specific loci included will be overestimated (Mak, Porsch, Choi, Zhou, & Sham, 2017). More sophisticated approaches (below) also need to deal with nonindependence of SNPs, usually by retaining all SNPs in the score while adjusting for LD in some way. Typically, LD is estimated from an external ancestry‐matched (as close as possible to the original GWAS super‐population; Figure 2C) reference panel (such as the 1000 genomes reference panel, Siva, 2008). However, the target sample can be used as the LD reference panel if the sample size is large enough (e.g. *N* > 1000; Vilhjálmsson et al., 2015) and provided it is representative of the GWAS super‐population.

C + T can be performed in PLINK (Purcell et al., 2007), but dedicated software, PRSice2, also exists (Choi & O'Reilly, 2019) that streamlines the procedure in memory and computationally efficient way. In addition, PRSice2 offers a ‘high‐resolution scoring’ option that permits to finetune PGS calculation across potentially hundreds of *p*‐value thresholds. Other methods to optimize C + T have been developed (Privé, Vilhjálmsson, Aschard, & Blum, 2019) extending the high‐resolution option idea of PRSice2 to other parameters (e.g. optimizing also with respect to clumping parameters). Advantages of the standard C + T method over others are computational efficiency, ease of use, and straightforward interpretation of the calculated score as the sum of included SNPs above a particular *p*‐value threshold, weighted by GWAS marginal effects.

### Polygenic score optimization – which SNPs to include

The standard GWAS threshold to select SNPs is often too restrictive for the purpose of PGS construction, and typically more lenient thresholds for SNPs inclusion in the score will lead to higher predictive power, depending on genetic architecture. This is often the case with highly polygenic traits, where a more liberal inclusion of SNPs in the score improves prediction. However, a PGS derived from all SNPs (i.e. with a *p*‐value threshold <1) could also be suboptimal due to added noise across many false positives SNPs included.

In practice, the optimal threshold for inclusion is unknown a priori and optimization with respect to *p*‐value thresholds (or tuning parameters for advanced approaches) should be performed to maximise accuracy. This, however, cannot be performed on the same sample where PGS effects are evaluated (i.e. the target set) because of overfitting. That is, the selected score will result in a prediction that will be too optimistic and will tend to underperform if applied to an external independent sample. Instead, an independent validation set (Figure 2D) will need to be used to select the best threshold (or tuning parameters of interest), which will then be employed in the external target set (or in the hold‐out set; Figure 2E).

Alternatively, tuning parameters can be optimized in the target set directly via cross‐validation, such as *k*‐fold repeated cross‐validation. However, to ensure generalizability, the performance of PGS should be tested in an external (or hold‐out) target set as the gold standard (Choi, Mak, & O’Reilly, 2020). Note that any bias in the sample (e.g. attrition), will reduce the performance of the (cross)validated model in independent data. That is, the validated model will not generalize well if the data used for validation is not representative of the target data. Finally, when large‐scale individual level‐data are available, solutions have been proposed to deal with the overlap between discovery‐target and validation‐target sets (Mak, Porsch, Choi, & Sham, 2018). Overlap between discovery and target sets can be addressed by cross‐prediction consisting in splitting the sample in *N* folds, estimating SNP effects in *N* − 1 folds, and conducting PGS analyses in the left‐out fold (Figure 2B). Overlap between the validation and target sets can be addressed by split validation, consisting in splitting the target sample into two parts, both of which are used in turn for validation and testing of the PGS.

In practice, often researchers may not have available samples large enough to validate their PGS in this way. Additionally, these PGS validation methods can be computationally expensive, a challenge that can be exacerbated especially when conducting already sophisticated longitudinal models. A solution to this problem can be to preselect one threshold (in the case of C + T) a priori. This can be done either based on previous evidence in a similar sample, or on prior assumptions such as the polygenicity of a trait (i.e. number of causal SNPs involved in the trait of interest). However, in practice, the optimal threshold is often not known a priori, and using a preselected threshold is likely to lead to underperformance of the PGS.

It is commonplace for researchers to report PGS results across an array of *p*‐value thresholds. However, interpretation based on the optimal PGS will be biased upwards and correction for multiple testing will need to be performed. One solution implemented in PRSice2 is to perform permutation tests and to use empirical *p*‐values as evidence for association. While this is appropriate to establish if an association exists, it will not correct for the aforementioned bias in effect sizes. Therefore, for PGS studies that involve interpretation of coefficients or assessing predictive accuracy, an independent out‐of‐sample or hold‐out set is recommended. An alternative solution consists of taking the first principal component out of a number of calculated PGS thresholds (Coombes, Ploner, Bergen, & Biernacka, 2020). By obtaining a unique score capturing the most variance across PGS thresholds, this approach gets around the validation/overfitting problem since PCA is an unsupervised method (i.e. it does not take into account the outcome of interest).

### Advanced methods for constructing PGS

Discussing all available methods to compute PGS is outside the scope of the present review, and we refer the reader to previous work comparing different PGS approaches across settings (Ni et al., 2021; Pain et al., 2021) and to the original PGS papers (Table S1). Here, we limit the review to the main features of PGS methods and their relative advantages.

Methods to construct PGS tend to vary depending on two broad themes: which SNPs are included in the scores, and the distribution from which SNP effect sizes are drawn. Depending on these, (re)weighting of SNP effect sizes from GWAS summary statistics is performed along with some form of shrinkage (the penalization of parameter estimates to improve accuracy, for example, based on LD between included SNPs). More generally, methods can be divided into Bayesian and frequentist approaches that differ in terms of how they attempt to model genetic architecture to improve prediction accuracy.

In practice, this translates into shrinking parameter estimates which typically improves predictions over marginal effects from GWAS because it reduces the total variance/noise in the estimates of the summed‐up SNPs. For example, thresholding in C + T can be thought of as a type of shrinkage, where certain effect sizes are shrunk exactly to 0. Different methods apply different types of shrinkage, and they will tend to perform favourably compared to others depending on the true underlying mixture of distributions of the trait of interest (Choi et al., 2020). The underlying trait distributions are in practice unknown, hence the optimal tuning parameters will need to be validated, as discussed above.

For example, methods such as SBLUP (Robinson et al., 2017), assume an infinitesimal model (akin to the ‘infinitesimal’ option in the method LDpred/LDpred2), where all SNPs are included in the scores and effect sizes are drawn from a normal distribution. This performs uniform shrinkage of the estimates across SNPs, adjusting for (local) linkage disequilibrium from a reference panel, effectively assuming that all SNPs have nonzero effects. That is, by assuming a uniform prior, effect sizes of causal variants are spread across neighbouring SNPs. This assumption may be problematic if the true underlying genetic architecture is sparse, for example, if only 5% rather than all the SNPs are causal (Vilhjálmsson et al., 2015). By contrast, the popular LDpred method and its extension, LDpred2, can accommodate noninfinitesimal genetic architectures by assuming a point‐normal mixture distribution for SNP effect sizes (Vilhjálmsson et al., 2015). Here, only a specific fraction of markers is assumed to be involved in the trait and drawn from a normal distribution, while the rest is fixed to 0.

Other Bayesian regression methods vary in terms of the shrinkage applied to SNP effect sizes and how, in turn, they handle different genetic architectures. Two examples of such methods are PRScs, which assumes a continuous shrinkage prior, robust to varying genetic architectures (Ge, Chen, Ni, Feng, & Smoller, 2019), and SbayesR (Lloyd‐Jones et al., 2019) which assumes that SNP effects sizes are drawn from a mixture of four distributions with mean 0 and different variances, whilst assuming varying contributions of SNPs coming from the different distributions (Ni et al., 2021).

Another type of shrinkage comes from the frequentist penalization method lassosum (Mak et al., 2017), where either a lasso (or L1 penalty, ${∥\beta ∥}_{1}$), or elastic net penalty, is applied on GWAS effect sizes. Frequentist penalization methods can be likened to Bayesian priors. For example, the lasso penalty can be likened to drawing effect sizes from a double exponential distribution effectively introducing sparsity, retaining only one effect size from a set of correlated SNPs, and shrinking the rest towards or exactly to 0 (Tibshirani, 1996).

All these approaches differ in terms of the assumed contribution of SNPs to the trait of interest. However, each approach implements a specific heritability model (Appendix S1) in that the same parameters (e.g., infinitesimal prior) are applied to every SNP. A novel prediction tool, MegaPRS (Zhang, Privé, Vilhjálmsson, & Speed, 2021), re‐implements a range of methods discussed above (with different software), but allows specifying parameters of prior distributions directly at the level of single SNPs (i.e. using different heritability priors for different SNPs). This allows for more realistic heritability models, in turn increasing the predictive power of PGS (Zhang et al., 2021).

Most of these methods require validation to optimize tuning parameters, with the exception of single score methods (e.g. infinitesimal models, Table S1). However, several approaches also offer a pseudovalidation (or automatic) option that discovers the optimal combination of tuning parameters automatically from the data, without an external validation sample. This can be advantageous for issues of sample splitting and power, as mentioned above, although pseudovalidated scores tend to perform less well compared to the optimized version using a validation sample (Yang & Zhou, 2022).

#### What method works best

In general, all advanced methods that directly account for LD tend to perform better than C + T (and variations thereof, Table S1) as less information is discarded across the genome. However, it is not entirely clear which of these methods performs best in different settings. Their performance is likely to depend on several factors including genetic architectures of discovery and target traits, tuning parameters, LD reference sample employed and statistics used to assess performance. To date, two studies have systematically benchmarked the prediction accuracy of PGS methods across a number of complex traits in either child or clinical samples.

One study comparing 10 PGS methods and focusing on the adult case–control psychiatric disorders from the Psychiatric and Genetic Consortium (PGC), including major depressive disorder (MDD) and Schizophrenia (SCZ) (Ni et al., 2021), highlighted the performance of SBayesR across settings. A second study comparing 8 methods focused instead on 4 continuous traits in adolescence and early adulthood from the Twin Early Development Study (Rimfeld et al., 2019), as well as 11 adult binary and continuous traits from the UKbiobank (Pain et al., 2021). In this study, LDpred2 performed best when parameter tuning was performed (but similarly to Lassosum and PRScs), while PRScs tended to perform best across scenarios in both adolescent and adult cohorts when using pseudovalidation. However, no dramatic differences were observed (on average) between advanced methods across different settings in both studies, although more nuanced results emerged depending on specific applications and settings (e.g. diverse genetic architectures).

While it remains difficult to choose an optimal method above all others, within specific settings more guided decisions can be made based on available evidence. If a validation cohort is available, then lassosum, PRScs, LDpred2 and MegaPRS are a good bet, with lassosum being the fastest computationally. If a validation cohort is not available, then SBayesR, PRScs, MegaPRS and LDpred2‐auto have comparable performance, with SbayesR being the fastest method computationally.

More recently a study comprehensively benchmarked the performance of 12 PGS methods across 50 adult complex traits (25 quantitative and 25 dichotomous) in the UKbiobank, extending PGS comparisons to different settings including cross‐ancestry performance (Yang & Zhou, 2022). Here, analyses showed that DBLSM (Yang & Zhou, 2020) tended to perform best across all settings, with the other two best‐performing methods (depending on the setting) being lassosum and LDpred2. In Table S1, we provide a comprehensive list of available methods and related tutorials.

#### Multitrait extensions

To improve the predictive power of PGS, the PGS framework can be extended to multitrait methods. In multitrait methods, the genetic correlation across traits are leveraged to improve the accuracy of SNP effect sizes and, hence, the predictive power of PGS. Generally speaking, multitrait approaches can be GWAS‐based methods where the focus is on detection of trait‐associated variants (e.g. MTAG and GenomicSEM, Grotzinger et al., 2019; Turley et al., 2018), and prediction‐based methods (e.g. SMTpred, Maier et al., 2018) where either GWAS summary statistics or PGS are combined in a weighted index. In both cases, the improved predictive power of PGS is achieved by obtaining optimal SNP weights from the combination of (genetically) correlated traits. We note that equivalent individual‐level methods exist (e.g. Maier et al., 2015; Pritikin, Neale, Prom‐Wormley, Clark, & Verhulst, 2021), with the caveat that for GWAS‐based methods relying on individual‐level data it is difficult to reach sample sizes as those based on summary‐level data. Previous work focusing on cognitive related traits across development showed that there might be optimal combinations between PGS approaches and multitrait methods in terms of predictive power (e.g. a combination between MTAG to obtain summary statistics and lassosum to compute the PGS, Allegrini et al., 2019). However, systematic evidence in this regard with respect to novel PGS methods and across different traits is currently lacking. Finally, it is possible to combine PGS for different traits in multivariable models, for example in penalized regression, to improve predictive power (Krapohl et al., 2018). This can be done also at the level of single PGS traits by combining different PGS thresholds, or PGS calculated using different tuning parameters, in the same model (Pain et al., 2021).

### Incorporating external (biological) information

As previously detailed, and further discussed in Appendix S1, the inaccurate estimation of SNP effects hampers PGS prediction. When added up to form a score, noise in estimated SNP effects builds up, yielding suboptimal PGS. We do not have information on the full set of causal variants involved in any given complex trait, but we can tag (some of) them with correlated genotyped and imputed SNPs. However, due to LD it is difficult to pinpoint causal variants as well as accurately estimate their effects (Hu, Lu, Powles, et al., 2017).

PGS can be extended to include external information, such as functional annotations, to improve prediction accuracy by prioritizing likely causal variants in the scores (Hu, Lu, Liu, et al., 2017; Hu, Lu, Powles, et al., 2017; Shi et al., 2016).

For example, LDpred‐func (Márquez‐Luna et al., 2021) builds on LDpred to include functional annotations in the prior used to reweight SNP effect sizes. This in turn yields improved PGS performance over a number of other PGS approaches (both annotation‐informed and not) for a number of traits (e.g. college education; Márquez‐Luna et al., 2021).

Another approach that has recently been proposed, PRS‐set (Choi et al., 2021), extends PRSice2 to include information on specific biological pathways (for example pathways implicated in neuronal function in individuals diagnosed with schizophrenia; Ripke, Walters, & O'Donovan, 2020). Where other approaches assume that people vary on a continuum from low to high polygenic burden for a particular trait, PRSset aims to capture heterogeneity in the polygenic signal by mapping SNPs to different biological pathways and functions. In practice, instead of creating one genome‐wide polygenic score for a particular trait, PRSset creates separate pathway‐PGS reflecting different biological processes or functions, which in turn can be employed for (disease) stratification and attempt to investigate biological relevance in complex traits. For example, schizophrenia‐based pathway‐PGS have been employed to uncover putative molecular mechanisms driving the association between schizophrenia polygenic risk and social behaviour in a child and adolescent sample (Schlag et al., 2021).

#### Repositories and resources

Several publicly available resources, including atlases, repositories and workflows, exist that can help researchers develop and implement PGS within a standardized framework (Table S2 provides references for a list of relevant resources). For example, GWAS summary statistics used to derive PGS can be retrieved from curated repositories such as the GWAS catalog (MacArthur et al., 2017), or Open GWAS (Elsworth et al., 2020), while atlases of GWAS and PGS results can be explored to inform analytical decisions. For example, the GWAS atlas (Watanabe et al., 2019) provides results of downstream analyses including SNP heritability and genetic correlations, that can be used to select appropriate summary statistics for particular PGS‐trait associations (e.g., screening summary statistics with highest genetic correlations with the trait of interest). The PGS atlas (Richardson, Harrison, Hemani, & Smith, 2019) reports phenome‐wide analyses of PGS for a wide array of traits that can be screened to, for example, prioritize certain traits in further analyses. Finally, GenoPred (Pain et al., 2021) provides a workflow for PGS analyses that can be employed to benchmark PGS performance across different methods and target sets.

There are also repositories of already developed PGS which allow researchers to construct PGS within a reproducible framework. For example, the PGS catalog (Lambert et al., 2021) reports SNPs, PGS weights and relevant metadata, including performance metrics, of published PGS. As such, researchers can employ PGS constructed in previously published research on their target sample, without having to use dedicated software to develop the score from scratch (Figure 2F). In a similar fashion, the PGI Repository (Becker et al., 2021) provides LDpred PGS weights for a number of complex traits in either single score or multitrait (MTAG) versions from a reference standardized pipeline. Furthermore, for a set of cohorts (https://www.thessgac.org/pgi‐repository) the PGI Repository provides already calculated single and multitrait scores based on GWAS including data from 23andMe, which is typically excluded from most PGS studies due to access restrictions.

## Applications to longitudinal designs

### Genetic continuity

In a typical setting, researchers test associations between PGS for adult traits with child phenotypes to infer continuity of genetic risk longitudinally. Equivalently, a recent study employed a PGS derived from a GWAS of childhood aggression to demonstrate genetic continuity of aggressive behaviour throughout the lifespan (Van der Laan et al., 2021). While the genetic code does not change throughout the lifespan, beta estimates used to construct PGS will capture average genetic effects on a phenotype that was collected at, and therefore is related to, a specific time and context. As such, the meaning of the PGS is heavily reliant on the phenotypic definition employed in GWAS. In this regard, different facets of the polygenic contribution (or liability) to a trait can be captured depending on the developmental period of interest, and on the SNP‐phenotype associations from which beta estimates to construct PGS are derived.

For example, by testing for an association between a PGS for adult body mass index (BMI) with childhood BMI, it is possible to infer genetic continuity of risk across the lifespan. However, the adult BMI PGS will likely become more predictive of the BMI phenotype at later stages in life, likely because the target trait becomes closer to the phenotype assessed in the original GWAS used to construct the PGS. Another plausible and nonmutually exclusive reason for this is (active) gene–environment correlation (Plomin, 2014; see Pingault et al., 2022). However, the adult BMI PGS is unlikely to capture the full complexities of childhood BMI across development, as highlighted in recent work (Helgeland et al., 2021). Based on a stratified BMI GWAS conducted across different developmental stages, BMI PGS were developed and tested for association with the corresponding developmental phenotypes. The predictive accuracy of PGS reflecting genetic influences on BMI at specific developmental stages was substantially greater compared to the PGS for adult BMI, and the pattern of associations highlighted developmental changes in the genetics of BMI. In a similar fashion, a PGS based on child case–control diagnosis of ADHD can improve our understanding of ADHD across child development, but misses the full (genetic) complexity of the disorder across the lifespan (Agnew‐Blais et al., 2021).

### Developmental stability and change

Stability and change across development can be investigated with latent growth curve (LGC) modelling techniques, by which we attempt to model between‐person (interindividual) differences and within‐person (intraindividual) changes over time (Curran, Obeidat, & Losardo, 2010). We can estimate LGC in a structural equation modelling (SEM) framework where we specify a latent (random) intercept and slope to capture, respectively, mean stable effects and mean change over time, as well as the variability around these (individual trajectories around linear or nonlinear changes). This is akin to fixed and random effects in a multilevel model. That is, we aim to measure random variability at the starting point (the intercept) and random variability in change (the slope).

Developmental stability and change have been extensively investigated in behavioural genetics, particularly with the use of twin studies. Studies employing PGS to investigate developmental questions inevitably build upon the rich behavioural genetics literature in this regard. With twin data, it is possible to estimate the contributions of genetics and the environment across development, including using LGC modelling (Neale & McArdle, 2000). For example, investigations of ADHD symptoms and conduct problems (Pingault, Rijsdijk, Zheng, Plomin, & Viding, 2015; Pingault, Viding, et al., 2015) have highlighted how different genetic factors contribute to baseline levels versus developmental change in these traits.

Findings from such twin studies can be followed up and expanded upon with the use of PGS. In conditional growth curve models, we can look at specific PGS predictors of this stability and change over time. Here, PGS can be modelled as time‐invariant covariates to predict the random variability component of the model. That is, whether a particular genetic predisposition for some trait associates with specific trajectories, the stable component or the rate of change over time. Recent work (Kwong et al., 2021) for example employed a multilevel random intercept and slope growth curve model to examine how PGS for adult psychiatric disorders is associated with developmental trajectories of depressive scores. Here, all psychiatric PGS were found to be associated with greater levels of depression throughout adolescence (i.e. with the intercept). However, only the depression‐related, and neuroticism PGS were found to be associated with a linear change of depression over time (the slope), as opposed to the schizophrenia and anxiety PGS for which no, or weak, evidence was found in this regard.

**Figure 3:**
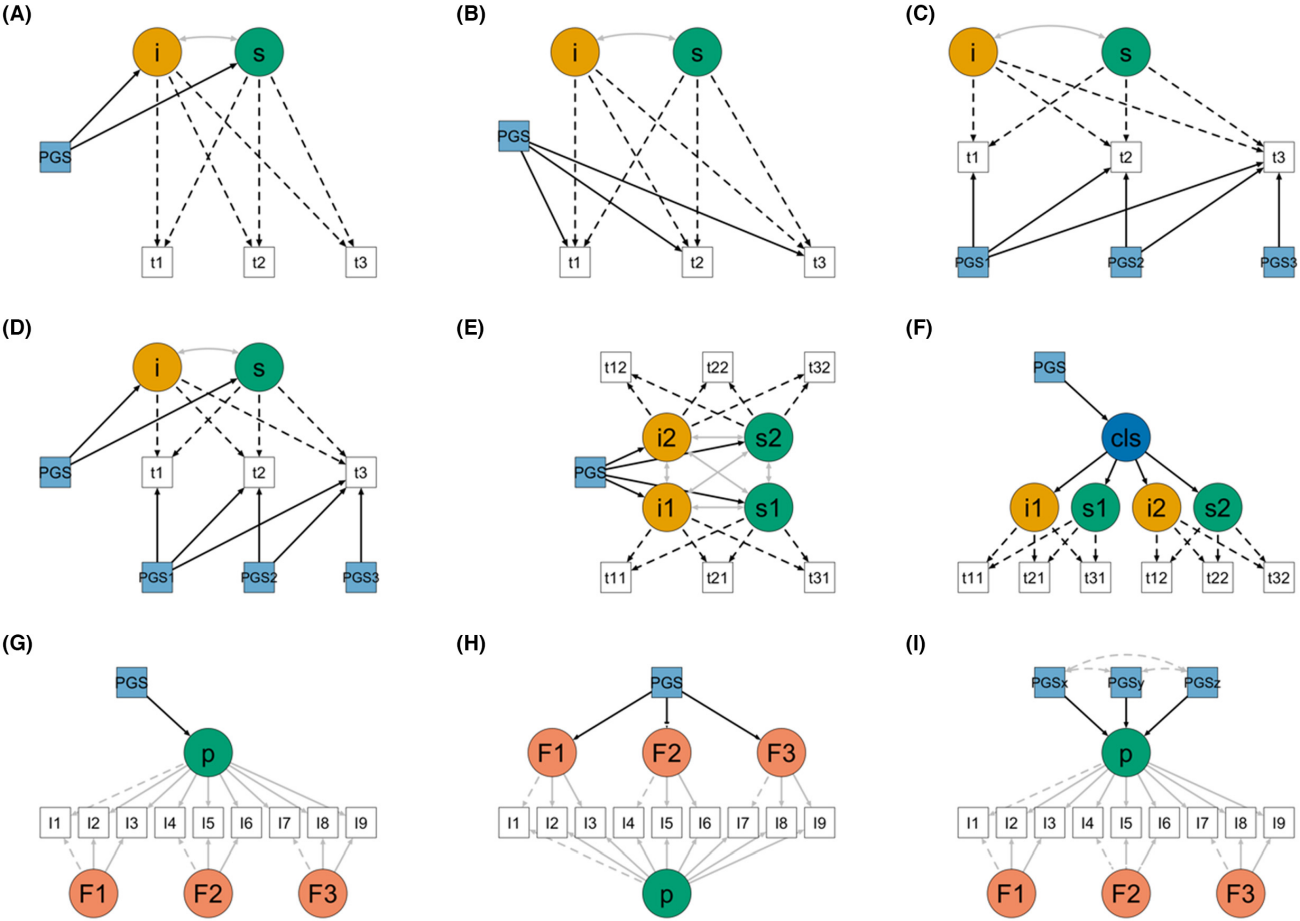
Different implementations of PGS in developmental models. Panels A to F, represent conditional latent growth curve models (LGC) and extensions thereof, with time‐invariant and time‐varying predictors. Panels G to I represent bifactor models. Note: residual variances and covariances are not shown for clarity. i = Intercept, s = slope, Cls = Class; *t*1–*t*3 = measurement at time 1–3; I1–I9 = Indicators 1–9. Figures were created based on Lavaan models, with the R package Semplot (Epskamp, 2015)

It is intuitive to conceptualize PGS as time‐invariant predictors since the DNA code does not change throughout the lifespan of an individual. It also makes sense in practice because often adequately powered GWAS are based on samples of adults (and often with restricted characteristics: limited to a particular ancestral, geographical, cultural and socioeconomic background). We can in turn ask whether PGS associate with some trait of interest via systematic developmental processes (prediction of latent growth factors – that is, intercept and slope) or age‐specific processes (prediction of age‐specific residuals) (Figure 3A,B). It is important to keep in mind, however, that such PGS reflect genetic influences on a trait that depends upon a specific phenotype definition, measured within a restricted age range and context.

As discussed, an alternative approach is to conduct repeated measures GWAS across development and in turn, construct PGS for specific developmental stages. These can be implemented as time‐varying covariates in longitudinal models such as LGC models, as shown in Figure 3C. For example, PGS for traits at specific developmental stages might differentially explain variability at certain occasions above and beyond the effects of the underlying trajectory, at the level of both contemporaneous and lagged effects. We can extend this idea in many interesting ways. For example, time‐varying predictors (e.g. lifestyle or risk factors) could be included in the model to investigate interactions between the PGS and such time‐varying covariates. In the case of the time‐varying PGS, we could impose equality constraints to test whether these PGS effects are constant over time, or whether their influence increases or decreases across development. Finally, we could conceptualize an ‘unspecific’ PGS as a time‐invariant predictor influencing the trajectories, and time‐specific PGS as time‐varying predictors contributing to the residual variation at each specific occasion (Figure 3D).

This framework can be extended to examine the association between PGS and joint trajectories, using a multivariate LGC (Figure 3E). Similarly, latent class trajectory models (LCGC) can capture putative latent groups in the trajectories, for example, an early onset vs late‐onset group (see Herle et al., 2020 for a review of such methods). For example, Hannigan et al. (2018) modelled the codevelopment of conduct and emotional problems in childhood using a joint trajectory latent class growth model. After identifying distinct joint trajectory classes of codeveloping emotional and conduct problems, PGS were used to predict membership to such classes. Specifically, the PGS for depression and educational attainment were found to predict respectively increased and decreased likelihood of belonging to the higher severity classes (Figure 3F).

### Heterogeneity and specificity across development

Polygenic scores can also be used to examine the specificity of genetic influences on developmental outcomes. That is, do PGS effects vary depending on particular domains, such as the age of assessment? A multivariate metaanalysis can answer such questions by testing for heterogeneity of effects across different domains. For example, Nivard et al. (2017) implemented a metaregression to demonstrate that the effects of a polygenic score for schizophrenia on measures of child psychopathology increased with age. However, the strength of association and the age‐related increases depended on the specific disorder considered. Similarly, Schlag et al. (2021) found differential effects of PGS for psychiatric traits on social behavioural phenotypes (e.g. peer problems), as well as age‐moderated effects, depending on the behavioural problem subtype considered. This type of analysis can shed light on differences in polygenic contributions to complex traits across development.

### Phenotypic stability and specificity

Twin studies have shown how substantial genetic effects underlie trait stability over time (e.g. Lubke et al., 2016). It has recently been shown (Cheesman et al., 2017; Gidziela et al., 2021) that by modelling stability it is possible to improve polygenic prediction of psychopathology traits across development as well as (and as a consequence of) increasing SNP‐*h* ^2^ (Appendix S1). To investigate phenotypic stability, an SEM framework can be used to create (latent) composites of traits across different domains (e.g., measures, raters and/or time) reducing error and capturing shared variance across domains. Twin and family studies have highlighted how a single genetic dimension partly underlies diverse disorders (Lichtenstein et al., 2009; Pettersson, Anckarsäter, Gillberg, & Lichtenstein, 2013; Pettersson, Larsson, & Lichtenstein, 2016). Similar investigations across childhood have pointed to substantial genetic contributions to the general psychopathology factor (*P*), as well as substantial genetic stability of P across time (Allegrini et al., 2020; Avinun, Knafo‐Noam, & Israel, 2021). Such investigations rely on modelling common and specific psychopathology dimensions with hierarchical models, such as second‐order and bifactor models (Figure 2G–H). Initial findings from the twin literature can be enriched by PGS‐based investigations, as detailed below.

The factors obtained in hierarchical models can in turn be related to PGS for an array of traits, either in univariate or multivariable modelling such as in multiple indicator and multiple causes models (Figure 3I). This allows investigation into whether the genetic liability of particular traits acts via common or specific factors, or whether these uniquely contribute to some indicators. Some of the work discussed in this area is cross‐sectional, but hierarchical methods can be naturally extended to the longitudinal case (Caspi et al., 2014). For example, an investigation of genetic contributions to psychopathology in childhood found that PGS effects over child behaviour problems were largely nonspecific, mediated by both general and specific, or only general, dimensions (Neumann et al., 2020; Riglin et al., 2020). A different, but related way to investigate polygenic risk and phenotypic specificity is to test for association with a PGS after adjusting for latent factors, for example, by testing associations of specific factors with a PGS after adjusting for the common factor (Waszczuk et al., 2021).

Another way to look at stability is by combining PGS in a unique measure of polygenic liability via PCA, and relating it to latent scores of (general) psychopathology (Allegrini et al., 2020). This can be employed in multiple ways to investigate specificity. For example, Morneau‐Vaillancourt et al. (2021) looked at associations between different PGS for mental health traits and a general mental health PGS with trajectories of social withdrawal. The general mental health PGS and the PGS for loneliness differentially predicted class membership to social withdrawal trajectories, uncovering specificity at the level of polygenic predisposition. Finally, an elegant way to look into the problem of specificity of PGS effects in childhood psychopathology has been proposed by Hannigan et al. (2021). Here, a model fit comparison of nested bifactor models allowing for effects of an SCZ PGS on respectively common (general) vs (domain) specific dimensions vs item‐level (residual) indicators was conducted. This allowed uncovering symptom‐specific effects of the SCZ PGS, in turn highlighting substantial heterogeneity in polygenic contributions within psychopathology dimensions.

## Summary

The increasing availability of genotype data in population and family based longitudinal samples allows for powerful applications of PGS to investigate and expand on developmental questions traditionally addressed by twin designs. Furthermore, ongoing efforts to standardize workflows and data repositories allow for a reproducible open‐science framework, fostering replicability.

We provided a general overview of PGS methodology, from theory to implementation in longitudinal designs, highlighting avenues and relevant resources. We highlighted applications of PGS in developmental models as they are most commonly employed in the literature, and how it can be further extended in future work. However, this is not an exhaustive list of all possible applications of PGS to developmental, longitudinal, designs. A number of longitudinal models not discussed here hold promise for future PGS work (Herle et al., 2020; Mund & Nestler, 2019). Of particular interest is the implementation of PGS in cross‐lagged designs allowing for longitudinal relationships between measurement occasions while modelling stability and change (Mund & Nestler, 2019).

Future work should also focus on modelling stability, change and specificity directly at the GWAS level by leveraging multivariate GWAS approaches. This can be done both in terms of individual‐level data, as genotype data from large longitudinal cohorts becomes available, or summary‐level data, as stratified GWAS become increasingly available (e.g. Ip et al., 2021; Jami et al., 2021). For example, a LGC model could be fit to the data to then conduct a GWAS of the slope (Pritikin et al., 2021), reflecting changes in developmental traits. Summary statistics thus obtained could in turn be employed to construct a polygenic score to be implemented in longitudinal designs.

Advances in PGS methods are especially important for childhood (psychopathology) phenotypes where the power of PGS is typically reduced, given that large GWASs are generally based on adult outcomes. Performance of different methods is variable, especially depending on the trait of interest if misspecification of underlying architecture occurs, but often not dramatic. Workflows, and atlases benchmarking performance of PGS and PGS methods across an array of traits, have an important role to guide researchers in the choice of methods.

Finally, the rapid advancement of the behavioral genetics field, both in terms of methods and (GWAS) results, is one challenge faced by repositories and other open resources, such as online tools, which, if not continuously maintained, run the risk of becoming rapidly outdated. One solution is afforded by community‐based repositories (e.g., PGS repository), and platforms (e.g., github), which can rely on users to stay up to date. Capitalizing on these advances will enable powerful and novel research applications to better integrate polygenic scores in child developmental psychology and psychopathology.

## Supporting information


**Appendix S1**. Assessing the predictive power of PGS.
**Table S1**. PGS methods and related tutorials.
**Table S2**. General resources for PGS workflow.
