# Supplementary material for: Research Review: A guide to computing and implementing polygenic scores in developmental research
Source: J Child Psychol Psychiatry. 2022 Mar 30;63(10):1111–24. doi: 10.1111/jcpp.13611 (PMC10108570; doi:10.1111/jcpp.13611)
Supplement: Supplementary file 1 — Appendix S1. Assessing the predictive power of PGS. Table S1. PGS methods and related tutorials. Table S2. General resources for PGS workflow. [file JCPP-63-1111-s001.docx]

**Supporting Information**

"Research Review: A guide to computing and implementing polygenic scores in developmental research"

Andrea G. Allegrini^1,2^, Jessie R. Baldwin^1,2^, Wikus Barkhuizen^1^ & Jean-Baptiste Pingault^1,2^

^1^ Department of Clinical, Educational and Health Psychology, Division of Psychology and Language Sciences, University College London, London, UK.

^2^ Social, Genetic and Developmental Psychiatry Centre, Institute of Psychiatry, Psychology and Neuroscience, King’s College London, London, UK.

**Contents**

Appendix S1……………………..…………………………………………………….………3

Table S1. PGS methods and related tutorials………………………………………………….6

Table S2. General resources for PGS workflow………………………………………………8

| **Appendix S1** |
| --- |
| **Assessing the predictive power of PGS**  ***SNP heritability***  SNP heritability (SNP-*h*^2^), the proportion of phenotypic variability explained by tagged variants, is by definition the ceiling of PGS prediction, as it represents the total contribution of all measured common variants jointly considered (Yang et al., 2010). SNP-*h*^2^ falls short of classic family-based narrow-sense heritability, in that SNP-*h*^2^ only measures additive genetic variation from common variants in unrelated individuals, and misses rare variation (not tagged by common SNPs) and structural variation that is shared within a family. In turn PGS explain only a small part of the SNP-*h*^2^. In this sense SNP-*h*^2^ is a useful metric to determine the limits of PGS.  ***Gap between PGS and SNP h^2^***  Estimated beta effects obtained from GWAS contain errors that sum up when combining SNPs within individuals. In contrast, SNP-*h*^2^ is obtained by jointly estimating independent SNP effects, using a mixed model approach in individual-level data among unrelated individuals (GCTA) (Yang et al., 2011). Alternatively, using summary-level data it can be approximated by regressing GWAS chi-square statistics on LD scores (LDSC) (Bulik-Sullivan et al., 2015). Hence PGS prediction is expected to be substantially lower than SNP-*h*^2^ unless effects sizes are estimated from massive sample sizes. That is the estimated $\hat{PRS}$ is different than the ‘true’ PRS due to imprecision in estimation (along with imperfect tagging of causal variants) (Wray et al., 2019).  It is important to highlight that specific methods, both at the PGS and SNP-*h*^2^ level, imply different heritability models (see section on advanced PGS methods). A different heritability model to compute SNP-*h*^2^ is implemented in the LDAK software (Speed et al., 2012), for individual-level data, and SumHer (Speed et al., 2020) software, for summary level data. Briefly, while the GCTA model assumes that heritability explained by SNPs is constant, LDAK assumes an heritability model where each SNP varies in their contribution to the trait depending on (local) LD and MAF (the BLD-LDAK model recommended by the authors further assumes that SNPs effects sizes depend on SNP annotations) (Speed et al., 2020, Speed et al., 2017)  ***Predictive accuracy of PGS and heterogeneity***  The following equation can be used to describe predictive power of PGS:  $R^{2}=\frac{h_{M}^{2}}{1+M/\left( Nh_{M}^{2} \right)} 1$  Here *M* is the number of SNPs independently contributing to the trait, *N* is the size of the GWAS sample and *h*^2^_M_ correspond to the SNP-*h*^2^ (Daetwyler et al., 2008, Wray et al., 2013). Here it can be seen that predictive accuracy of PGS substantially depends on GWAS sample size, as *N* increases to infinity the denominator becomes 1 and R^2^ approximates SNP h^2^. Also of note is that, with constant *N* and as *M* increases, R^2^ decreases. This is because as the number of causal SNPs involved in the trait increases also does the imprecision of estimating them. Some polygenic scores methods discussed in main text attempt to tackle this problem by trying to balance more effectively *M* vs SNP *h*^2^ by effectively reducing the number of estimated effect sizes (Wray et al., 2019; Wray et al., 2013). One way to do that is by incorporating prior information such as genomic annotations to bin variants, and regularization methods that introduce sparsity in the data (e.g. LDpred-funct).  An important caveat in the predictive power of PGS is represented by phenotypic heterogeneity, i.e. the discrepancy between phenotypic definitions in the discovery (GWAS) and target set. In the context of polygenic prediction heterogeneity can be quantified as the imperfect genetic overlap between the trait in the discovery set where we estimate SNP effect sizes and the trait in the target set (independent out-of-sample or hold-out), where performance of PGS is tested.  The following equation describes predictive power of PGS in this regard (de Vlaming et al., 2017):  $R^{2}=\frac{{r_{g}h}_{M}^{2}h_{M}^{2^{T}}}{h_{M}^{2}+M/N} 2$  Here $h_{M}^{2^{T}}$ is the target set trait SNP-*h*^2^, while $h_{M}^{2}$ is the SNP-*h*^2^ of the trait in the discovery set. *r_g_* represents the genetic correlation between the discovery and target set traits. If we measure the same trait in the discovery and target set, assuming equal SNP-*h*^2^, and *r_g_* of 1, then this equation corresponds to equation 1 above. In turn, as the phenotypic definitions between the discovery and target set diverge, R^2^ increases as a function of *r_g_*_,_ with the upper bound of R^2^ given by ${r_{g}h}_{M}^{2^{T}},$as the discovery sample size increases (de Vlaming et al., 2017). When generalized to cross-trait prediction, this implies that it is possible to obtain higher PGS predictions cross-trait than within-trait, depending on the relative combinations of *r_g_* and target set SNP-*h*^2^.  **How to assess (PGS) prediction accuracy**  ***Continuous outcomes***  **-R^2^**: For continuous outcomes often researchers optimizing their model in a validation set will go on to calculate prediction accuracy as the squared correlation between predicted and observed values in the target set.  R^2^ = cor(predicted,observed)^2^  However, this is problematic as it allows for recalibration of the predictors in the target set, making R^2^ biased upwards. For example, if predictions in the target set are off by some constant, the correlation coefficient will not penalize for this, estimating a new slope and intercept on top of the predictions (Harrel, 2015). Instead, the correct formula that does not allow for internal recalibration is: R^2^ = 1 - SSE/SST. Where SEE = sum of squared error, and SST = sum of square total.  **-R^2^ difference:** A second way is to calculate prediction increase over a ‘null’ model. First a null model is fitted including only relevant covariates (e.g. sex + age + genetic PCs), then the same model if fitted adding the PGS as predictor. The difference in R^2^ between these models can then be calculated.  ***Case/control outcomes***  **-Nagelkerke’s R^2^:** equivalent to R^2^, but for case control outcomes.  **-R^2^ on the liability scale:** Nagelkerke’s R^2^ it’s a useful metric to compare different models in the same sample. However, as this metric relies on the proportion of cases, then R^2^ on the liability scale (Lee et al., 2012) is more appropriate for comparing models across samples with different case/control ratios (Wray et al., 2021).  **-AUC:** the area under the ROC curve is an index ranging from .5 to 1. It is the probability that a person randomly drawn from the cases will score higher than another randomly picked person for the control group. With a score of .5 indicating random accuracy and 1 perfect accuracy. |

| **Table S1.** PGS methods and related tutorials | | | | |
| --- | --- | --- | --- | --- |
| **Method** | **Software/extensions** | **Parameter tuning required** | **Single score/infinitesimal/auto option** | **Tutorial/Web/github** |
| **C+T** | Plink | Yes | No | - Web: <https://zzz.bwh.harvard.edu/plink/profile.shtml> - Tutorial: <https://choishingwan.github.io/PRS-Tutorial/plink/> |
|  | PRSice2 | Yes | No | - Tutorial: <https://choishingwan.github.io/PRS-Tutorial/> |
|  | Stacked-Clumping and thresholding | Yes | No | - Tutorial: <https://privefl.github.io/bigsnpr/articles/SCT.html> |
|  | PRS-PC | No | Default | - Web: [gepi22339-sup-0001-Supplemental_Material.docx](https://onlinelibrary.wiley.com/action/downloadSupplement?doi=10.1002%2Fgepi.22339&file=gepi22339-sup-0001-Supplemental_Material.docx) |
|  | PRSset | Yes | No | - Github: <https://github.com/choishingwan/PRSice/blob/master/docs/prset_detail.md> |
| **LDpred** | LDpred | Yes | Yes | - Github: <https://github.com/bvilhjal/ldpred> |
|  | LDPred2 | Yes | Yes | - Tutorial: <https://privefl.github.io/bigsnpr/articles/LDpred2.html> - Tutorial: <https://choishingwan.github.io/PRS-Tutorial/ldpred/> |
|  | LDPred-func | Yes | Yes | - Github: <https://github.com/carlaml/LDpred-funct> |
| **Lassosum** | lassosum | Yes | Yes | - Tutorial: <https://choishingwan.github.io/PRS-Tutorial/lassosum/> - Github: <https://github.com/tshmak/lassosum> |
|  | lassosum2 | Yes | Yes | - Tutorial: <https://privefl.github.io/bigsnpr-extdoc/polygenic-scores-pgs.html> |
| **SBLUP** | GCTA | No | Default | - Web: <https://cnsgenomics.com/software/gcta/#SBLUP> - Tutorial: <https://mobile.twitter.com/dr_appie/status/1382313130894721027> |
| **PRS-CS** | PRS-CS | Yes | Yes | - Github: <https://github.com/getian107/PRScs> |
| **SbayesR** | GCTB | No | Default | - Tutorial: <https://cnsgenomics.com/software/gctb/#SummaryBayesianAlphabet> |
| **MegaPRS** | LDAK | Yes | Yes | - Tutorial: <https://dougspeed.com/megaprs/> |
| **DBSLMM** | DBSLMM | No | Default | - Tutorial: <https://biostat0903.github.io/DBSLMM/> - Github: <https://github.com/biostat0903/DBSLMM> |

| **Table S2.** General resources for PGS workflow | | | |
| --- | --- | --- | --- |
| **Resource** | **Reference** | **Brief description** | **link** |
| Polygenic score catalog | (Lambert et al., 2021) | Database of PGS employed in published work providing relevant metadata to develop and evaluate them in different datasets. | <https://www.pgscatalog.org> |
| Polygenic index repository | (Becker et al., 2021) | PGS repository providing metadata to reproduce PGS, or already constructed PGS for a number of cohorts. PGS are obtained from a reference standardized and optimized pipeline. | <https://www.thessgac.org/pgi-repository> |
| Open GWAS | (Elsworth et al., 2020) | A curated collection of GWAS summary statistics. | <https://gwas.mrcieu.ac.uk> |
| GWAS catalog | (MacArthur et al., 2017) | A curated catalog of GWAS results and summary statistics. | <https://www.ebi.ac.uk/gwas/> |
| GWAS Atlas | (Watanabe et al., 2019) | Database of GWAS results and downstream analyses. | <https://atlas.ctglab.nl> |
| PGS Atlas | (Richardson et al., 2019) | Atlas of PGS – phenotype associations across 162 PGS and 551 traits. | <http://mrcieu.mrsoftware.org/PRS_atlas/> |
| GenoPred | (Pain et al., 2021) | A workflow for evaluating PGS methods within a reference standardized framework. | <https://opain.github.io/GenoPred/>  <https://github.com/opain/GenoPred/tree/master/GenoPredPipe> |

**References**

BECKER, J., BURIK, C. A., GOLDMAN, G., WANG, N., JAYASHANKAR, H., BENNETT, M., BELSKY, D. W., LINNÉR, R. K., AHLSKOG, R. & KLEINMAN, A. (2021). Resource profile and user guide of the Polygenic Index Repository. *Nature human behaviour***,** 1-15.

BULIK-SULLIVAN, B., FINUCANE, H. K., ANTTILA, V., GUSEV, A., DAY, F. R., LOH, P.-R., DUNCAN, L., PERRY, J. R., PATTERSON, N. & ROBINSON, E. B. (2015). An atlas of genetic correlations across human diseases and traits. *Nature genetics,* 47**,** 1236.

DAETWYLER, H. D., VILLANUEVA, B. & WOOLLIAMS, J. A. (2008). Accuracy of predicting the genetic risk of disease using a genome-wide approach. *PloS one,* 3**,** e3395.

DE VLAMING, R., OKBAY, A., RIETVELD, C. A., JOHANNESSON, M., MAGNUSSON, P. K., UITTERLINDEN, A. G., VAN ROOIJ, F. J., HOFMAN, A., GROENEN, P. J. & THURIK, A. R. (2017). Meta-GWAS Accuracy and Power (MetaGAP) calculator shows that hiding heritability is partially due to imperfect genetic correlations across studies. *PLoS genetics,* 13.

ELSWORTH, B. L., LYON, M. S., ALEXANDER, T., LIU, Y., MATTHEWS, P., HALLETT, J., BATES, P., PALMER, T., HABERLAND, V. & SMITH, G. D. (2020). The MRC IEU OpenGWAS data infrastructure. *bioRxiv*.

LAMBERT, S. A., GIL, L., JUPP, S., RITCHIE, S. C., XU, Y., BUNIELLO, A., MCMAHON, A., ABRAHAM, G., CHAPMAN, M. & PARKINSON, H. (2021). The Polygenic Score Catalog as an open database for reproducibility and systematic evaluation. *Nature genetics,* 53**,** 420-425.

LEE, S. H., GODDARD, M. E., WRAY, N. R. & VISSCHER, P. M. (2012). A better coefficient of determination for genetic profile analysis. *Genetic epidemiology,* 36**,** 214-224.

MACARTHUR, J., BOWLER, E., CEREZO, M., GIL, L., HALL, P., HASTINGS, E., JUNKINS, H., MCMAHON, A., MILANO, A. & MORALES, J. (2017). The new NHGRI-EBI Catalog of published genome-wide association studies (GWAS Catalog). *Nucleic acids research,* 45**,** D896-D901.

PAIN, O., GLANVILLE, K. P., HAGENAARS, S. P., SELZAM, S., FÜRTJES, A. E., GASPAR, H. A., COLEMAN, J. R., RIMFELD, K., BREEN, G. & PLOMIN, R. (2021). Evaluation of polygenic prediction methodology within a reference-standardized framework. *PLoS genetics,* 17**,** e1009021.

RICHARDSON, T. G., HARRISON, S., HEMANI, G. & SMITH, G. D. (2019). An atlas of polygenic risk score associations to highlight putative causal relationships across the human phenome. *Elife,* 8**,** e43657.

SPEED, D., CAI, N., JOHNSON, M. R., NEJENTSEV, S. & BALDING, D. J. (2017). Reevaluation of SNP heritability in complex human traits. *Nature genetics,* 49**,** 986-992.

SPEED, D., HEMANI, G., JOHNSON, M. R. & BALDING, D. J. (2012). Improved heritability estimation from genome-wide SNPs. *The American Journal of Human Genetics,* 91**,** 1011-1021.

SPEED, D., HOLMES, J. & BALDING, D. J. (2020). Evaluating and improving heritability models using summary statistics. *Nature genetics,* 52**,** 458-462.

WATANABE, K., STRINGER, S., FREI, O., MIRKOV, M. U., DE LEEUW, C., POLDERMAN, T. J., VAN DER SLUIS, S., ANDREASSEN, O. A., NEALE, B. M. & POSTHUMA, D. (2019). A global overview of pleiotropy and genetic architecture in complex traits. *Nature genetics,* 51**,** 1339-1348.

WRAY, N. R., KEMPER, K. E., HAYES, B. J., GODDARD, M. E. & VISSCHER, P. M. (2019). Complex trait prediction from genome data: contrasting EBV in livestock to PRS in humans: genomic prediction. *Genetics,* 211**,** 1131-1141.

WRAY, N. R., LIN, T., AUSTIN, J., MCGRATH, J. J., HICKIE, I. B., MURRAY, G. K. & VISSCHER, P. M. (2021). From basic science to clinical application of polygenic risk scores: a primer. *JAMA psychiatry,* 78**,** 101-109.

WRAY, N. R., YANG, J., HAYES, B. J., PRICE, A. L., GODDARD, M. E. & VISSCHER, P. M. (2013). Pitfalls of predicting complex traits from SNPs. *Nature reviews genetics,* 14**,** 507-515.

YANG, J., BENYAMIN, B., MCEVOY, B. P., GORDON, S., HENDERS, A. K., NYHOLT, D. R., MADDEN, P. A., HEATH, A. C., MARTIN, N. G. & MONTGOMERY, G. W. (2010). Common SNPs explain a large proportion of the heritability for human height. *Nature genetics,* 42**,** 565-569.

YANG, J., LEE, S. H., GODDARD, M. E. & VISSCHER, P. M. (2011). GCTA: a tool for genome-wide complex trait analysis. *The American Journal of Human Genetics,* 88**,** 76-82.
